# Clinical efficacy of motor imagery therapy based on fNIRs technology in rehabilitation of upper limb function after acute cerebral infarction

**DOI:** 10.12669/pjms.38.7.5344

**Published:** 2022

**Authors:** Yi Dai, Fang Huang, Yanfang Zhu

**Affiliations:** 1 Yi Dai, Department of Rehabilitation Clinic, Wuhan Puren Hospital, Wuhan 430080, Hubei, China; 2 Fang Huang, Department of Rehabilitation Clinic, Taikang Tongji (Wuhan) Hospital, Wuhan 430000, Hubei, China; 3 Yanfang Zhu, Department of Respiratory Medicine, Xiantao First People’s Hospital Affiliated, Xiantao 433000, Hubei, China

**Keywords:** Functional near-infrared spectroscopy, Motor imagery therapy, Cerebral infarction, Upper limb function, Rehabilitation

## Abstract

**Objective:**

To investigate the efficacy of motor imagery therapy (MIT) based on functional near-infrared spectroscopy (fNIRs) technology in rehabilitation of upper limb function after cerebral infarction, so as to provide guidance for clinical practice.

**Methods:**

A total of 84 patients with upper limb dysfunction after acute cerebral infarction admitted to Wuhan Puren Hospital from January 2020 to January 2021 were included in this study and randomly divided into two groups based on random number table method: experimental group and control group, with 42 cases in each group. Both groups were given active symptomatic treatment. The control group received conventional exercise rehabilitation, while the experimental group also received MIT based on fNIRs technology in addition to the treatment method adopted by the control group, lasting for eight weeks. The simplified Fugl-Meyer scale was utilized to evaluate the recovery of upper limb function, and the changes of Oxy-Hb and Deoxy-Hb concentrations in the frontal area of brain tissue were measured to evaluate the total effective rate of clinical rehabilitation.

**Results:**

At four and eight weeks of treatment, the Fugl-Meyer scores of the two groups were significantly higher than those before treatment (P<0.05), and those in experimental group were significantly higher than those in control group (P<0.05), with no significant difference before treatment (P>0.05). After treatment, Oxy-Hb concentration in the experimental group was higher than that in the control group (P<0.05), while Deoxy-Hb concentration was lower than that in the control group, with a statistical significance (P<0.05). The total effective rate of rehabilitation in the experimental group was 88.10%, which was significantly higher than 73.81% in the control group (P<0.05).

**Conclusion:**

Motor imagery therapy (MIT) Based on fNIRs technology has important clinical value in rehabilitation of upper limb function after cerebral infarction and is superior to conventional exercise rehabilitation alone, boasting a variety of effects, such as improving the curative effect, ameliorating blood oxygen in brain tissue, and promoting the rehabilitation of upper limb function of patients.

***Randomized Controlled Trial:*** RCT Registration Number is ChiCTR1800015546.

## INTRODUCTION

Limb motor dysfunction is a common symptom after cerebral infarction, especially upper limb dysfunction, which is very challenging in rehabilitation treatment.^1^ In the wake of the continuous improvement of clinical diagnosis and treatment in recent years, the mortality rate of cerebral infarction has been reduced to a certain extent, but the disability rate is still hovering at a high level.^2^ According to statistics, about 80% of patients with cerebral infarction have limb movement dysfunction of varying degrees, of which the upper limbs account for more than 60%, which not only greatly affects the health and life quality of patients, but also increases the consumption of clinical medical resources.^3^

Currently, the preferred clinical rehabilitation methods for upper limb dysfunction include double upper limb exercise training, rehabilitation robot, electrical stimulation, electromyography biofeedback, transcranial magnetic stimulation, etc., among which transcranial magnetic stimulation (TMS) has gradually become an important method for the treatment of upper limb motor dysfunction in patients with cerebral infarction in recent years. Existing studies have shown that performing TMS several times on patients with cerebral infarction contributes to inhibiting the abnormally high excitement of the motor cortex of the patient’s non-affected hemisphere, thereby promoting the rehabilitation of the motor function of the affected upper limb. It has become a hot spot in clinical research to measure cerebral oxygen saturation and cerebral hemodynamics to master brain function by virtue of near-infrared spectroscopy (NIRS).^4^ NIRS has determined that the neurophysiology and energy basis of brain function is a nerve-vascular coupling mechanism, which activates changes in local cerebral blood flow and oxygen metabolism in the cerebral cortex, resulting in changes in blood oxygen concentration in the activated region. In this paper, a prospective controlled study was conducted to investigate the application effect referred of upper limb functional rehabilitation after cerebral infarction.

## METHODS

Randomized controlled trial was used in this study. A total of 84 patients with upper limb dysfunction after cerebral infarction admitted to Wuhan Puren Hospital from January 2020 to January 2021 were selected as subjects. The sample size required for each group was calculated by the formula 
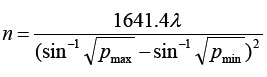
. All of them were diagnosed as cerebral infarction with upper limb dysfunction by symptoms and craniocerebral MRI.

###  Inclusion criteria:

- Patients who met the relevant diagnostic criteria for stroke and were confirmed to have upper limb dysfunction after a comprehensive examination;
- Patients with stable vital signs, clear consciousness, and able to cooperate with relevant examinations after cerebral infarction;
- Patients within 14 days of onset;
- Patients who themselves and their families know and agree to the study.

### Exclusion criteria:

- Patients with heart, liver and kidney dysfunction;
- Patients with a history of epilepsy;
- Patients with cognitive and communication disorders;
- Patients who wear a pacemaker and have metal implants in their bodies;
- Patients with deteriorating disease progression.

This study was conducted under the approval of the Ethics Committee of the hospital. All 84 patients were randomly divided into two groups by random number table method: experimental group and control group, with 42 cases in each group.

### Ethical Approval

The study was approved by the Institutional Ethics Committee of Wuhan Puren Hospital on January 2021 (No. [2021]05), and written informed consent was obtained from all participants.

### Methods

Both groups received symptomatic treatment, including activating blood, nourishing nerve and lowering blood pressure. Patients were followed up for rehabilitation after their vital signs were stabilized. Specifically, routine exercises were carried out for the control group, including limb placement, upper limb active and passive training, turning over training, sitting and standing training, and up and down stairs training, etc., under the guidance of professional rehabilitation physicians, 35-45min/d, 5d per week for 8 consecutive weeks.^5^

For the experimental group, MIT based on fNIRs technology was implemented in addition to the treatment taken by the control group. The FOIRE-3000 near-infrared functional brain imagery was used to construct a near-infrared three-dimensional brain function imaging map during motor imagery training, and then analyze the functional activity information of the cerebral cortex. In this way, patients were instructed to create a “flow chart” of the movement of the upper limb on the affected side and the sense of motor imagery in their head, and to rehearse the movements in memory repeatedly according to the instructions. Rehabilitation physicians were first asked to explain and demonstrate the movement essentials of motor imagery to patients, and then the patients rehearsed the movements of memory repeatedly in their hearts according to the guide language of motor imagery. All the imagery tasks applied in this study were selected from simple movements and upper limb movements in daily life, including the following aspects:

### (1) Guide language (two minutes):

Please close your eyes slowly, take a deep breath, relax your muscles, get rid of distracting thoughts, and think of yourself at the beach…… Follow the guidance to feel and experience each movement.

### (2) Motor im-1:

Please imagine that there is a cylindrical teacup on the table in front of you. Slowly stretch out your affected arm forward, loosen five fingers, and firmly grasp the teacup in front of you, slowly put it in your mouth to drink and then put it in place.

### Imagination-2

Please imagine that you are standing at the door of the ward with your affected hand grabbing the door handle and pushing the door open, then gently closing the door, and putting your arm back.

### Imagination-3

Please imagine that your affected hand with five fingers firmly stretched out, then make a fist with the greatest strength and raise it to show the physician your powerful strength.

### Imagination-4

Please imagine that you are sitting in the cab of a car with both hands on the steering wheel and the affected hand twisting the steering wheel vigorously.

### Imagination-5

Please imagine that you raise your hands and pick your favorite fruit, such as apples.

### Imagination-6

Please imagine using the fingers on the affected side to count, that is, use your thumb to pinch the other four fingers.

All the above actions were repeated 3 times.

### (3) Concluding remarks (2min):

Please open your eyes, take a deep breath, and relax your body slowly.

### Observation indexes:

***Motor function evaluation:*** The Fugl-Meyer scale was used to evaluate the upper limb function recovery of patients before treatment, at four and eight weeks of treatment respectively. There were 33 items in total, each with a score of 0-2, and a total of 66 points. A total score of < 33 was classified as severe dyskinesia, 33-55 as moderate dyskinesia, 56-60 as mild dyskinesia, and 61-66 as mild or no dyskinesia.^6^

### Hemoglobin index of brain tissue

Before treatment and 8 weeks after treatment, the concentration changes of oxygenated hemoglobin (Oxy-Hb) and deoxy-Hb (Deoxy-Hb) in the C4/C5/C14 channels in the frontal area of brain tissues of patients were detected by the fNIRs technology system.

### Efficacy evaluation

Evaluation was conducted according to the Fugl-Meyer scale after treatment: (1) *Cured*, Fugl-Meyer evaluation grade reached an independent status, and no recurrence was observed during follow-up; (2) *Markedly effective*, Fugl-Meyer evaluation grade increased by 2 or above; (3) *Effective*, Fugl-Meyer evaluation grade increased by 1; (4) *Invalid*, Fugl-Meyer evaluation grade has no change.^7^

### Statistical Analysis

SPSS22.0 was used for statistical analysis. Measurement data were expressed by mean ± standard deviation (*x̅*±*s*). The independent sample t test was used for comparison between groups, and paired sample t test was used for intra-group comparison. Moreover, measurement data were expressed by number of cases (%), and intra-group comparison was performed by X^2^ test. P<0.05 indicates a statistically significant difference.

## RESULTS

Twenty-three males and 19 females were enrolled in the experimental group, aged from 58 to 71 years, with an average of (62.52±5.11) years. The duration of upper limb dysfunction ranged from 7 to 25d, with an average of (14.20±3.11)day. Twenty-two males and 20 females were included in the control group, aged from 61 to 70 years, with an average of (63.46±5.05) years. The duration of upper limb dysfunction ranged from 10 to 23d, with an average of (14.13±3.15)d. No significant difference was observed in the basic clinical conditions of the two groups (P>0.05), indicating good comparability.

At four and eight weeks of treatment, the Fugl-Meyer scores of the two groups were significantly higher than those before treatment (P<0.05), and those in experimental group were significantly higher than those in control group (P<0.05), with no significant difference between the groups before treatment (P>0.05), as shown in Table-I.

**Table-I:** Comparison of Fugl-Meyer scores between the two groups before and after treatment (*x̅*±*s* points).

Group	Number of cases	Before treatment	4 weeks of treatment	8 weeks of treatment
Experimental group	42	36.44±9.16	50.12±8.55^*^	59.21±8.16^*#^
Control group	42	37.18±9.43	45.08±9.22^*^	52.30±8.64^*#^
t		0.914	10.362	11.115
P value		0.078	0.000	0.001

***Note***: Comparison between the same group and before treatment,

* P<0.05; Comparison between the same group and treatment for 4 weeks,

# P<0.05.

After treatment, Oxy-Hb concentration in the experimental group was higher than that in the control group (P<0.05), while Deoxy-Hb concentration was lower than that in the control group, with a statistical significance (P<0.05), as shown in Table-II.

**Table-II:** Comparison of hemoglobin concentration in brain tissues between the two groups before and after treatment (*x̅*±*s*, mmol/L).

Group	No. of cases	Oxy-Hb		Deoxy-Hb	

		Before treatment	After treatment	Before treatment	After treatment
Experimental group	42	0.01023±0.00091	0.01932±0.00090^*^	0.01002±0.00042	0.00224±0.00060^*^
Control group	42	0.01051±0.00093	0.01782±0.00086^*^	0.00928±0.00051	0.00562±0.00083^*^
t		1.04	4.34	0.91	3.10
P value		0.08	0.00	0.10	0.00

***Note:*** Comparison between the same group and before treatment,

* P<0.05.

According to the evaluation after treatment, the total effective rate of rehabilitation in the experimental group was 88.10%, which was significantly higher than 73.81% in the control group (P<0.05), with a statistically significant difference (P<0.05).

## DISCUSSION

Recovery of upper limb function after cerebral infarction is an intractable aspect of clinical rehabilitation owing to the fact that the area representing the motor function of the upper limb in the cerebral motor cortex is nearly twice that of the lower limb. Based on this, greater connections are required for peripheral nerve cell reconstruction after brain damage to promote the recovery of upper limb function.^8^ In the wake of the continuous development of rehabilitation medicine in recent years, rehabilitation therapy methods have been innovated and enriched. Among them, MIT, a new means of rehabilitation, has shown satisfactory results in the practice of clinical rehabilitation of limb function in patients with hemiplegia.

The so-called motor imagery is to train personnel to conduct repeated simulated exercise of motor behaviors in their mind without significant physical activities. In the field of rehabilitation therapy, detailed exercise can contribute to repairing or rebuilding damaged motor nerve conduction pathways, so as to awaken dormant synapses and play a compensatory role.^9^,^10^ In the early stage of cerebral infarction, patients can perform repeated motor imagery training to better ameliorate the function of the injured limb. In recent years, brain-computer interface (BCI) technology has been applied in the rehabilitation of patients with dyskinesias, through which patients with cerebral infarction are able to interact with the environment via brain signals instead of muscles, and promote motor function recovery through induced activity dependent on brain plasticity.^11^ fNIRs, as a dynamic detection of nerve cell activity technology, can realize a new neuroimaging technology for brain function detection, with the advantages of non-invasive, unaffected by motion artifacts and continuous detection. Motor imagery is an important method in the research field of BCI, while fNIRs technology boasts of measuring signal changes in oxygenated hemoglobin and deoxygenated hemoglobin from specific areas of the cerebral cortex during motor imagery, so as to carry out rehabilitation in a better way.^12^ It was reported in a study^13^ that MIT can effectively stimulate the cerebral cortex motor network of patients with cerebral infarction, and improve their impaired motor function by virtue of repeated motor imagery training.

**Table-III:** Comparison of the total effective rate of clinical rehabilitation between the two groups (n, %).

Group	Number of cases	Cured	Markedly effective	Effective	Invalid	Total effective
Experimental group	42	7 (16.67)	13 (30.95)	17 (40.48)	5 (11.90)	37 (88.10)
Control group	42	3 (7.14)	8 (19.05)	20 (47.62)	11 (26.19)	31 (73.81)
X^2^						14.033
P value						0.001

Traditional rehabilitation therapies are reported to have a preferable effect on the rehabilitation of limb dysfunction in patients with cerebral infarction, but its disadvantages, such as the need for one-on-one training and the failure of smooth development in the early stage, may have a negative impact on the effect to a certain extent.^14^ MIT, by contrast, is characterized by simple operation, early intervention and easy to master. Patients can train by themselves after receiving relevant guidance at the early stage of MIT implementation, which can greatly save time, improve compliance and enhance rehabilitation effect.^15^

In this study, the application of MIT in the rehabilitation of upper limb dysfunction after cerebral infarction was studied based on fNIRs technology. As indicated by the results, the FUGL-Meyer score of the experimental group was significantly higher than that of the control group at 4 and 8 weeks of treatment (P<0.05), and the total recovery rate of the experimental group was 88.10%, which was higher than 73.81% in the control group (P<0.05). The above results are basically consistent with those reported by relevant studies.^16^-^18^ It can be seen that MIT can promote the rehabilitation of upper limb function of patients after cerebral infarction and enhance the clinical efficacy.^19^ Meanwhile, changes in relative concentrations of OXY-Hb and DeOXY-Hb in the cortex of the relevant brain area before and after treatment were measured by fNIRs technique in this study. It has been shown in this study that with the increase of neuronal activity in cerebral cortex, the demand for energy metabolism of cells will also increase, followed by the increase of local oxygen consumption and craniocerebral blood flow, resulting in the increase of Oxy-Hb and the decrease of Deoxy-Hb. From the perspective of quantitative analysis, the concentration of OXY-Hb in the experimental group was higher than that in the control group, while the concentration of DeOXY-Hb was lower than that in the control group (P<0.05), suggesting that the application of conventional motor imagery therapy could increase the activity of corresponding neurons in the cerebral cortex and increase cerebral blood flow, resulting in the increase of Oxy-Hb and the decrease of Deoxy-Hb.

### Limitations of this study

The number of subjects included in this study was limited, so the conclusions drawn may not be very convincing. In addition, we only analyzed and discussed the cases included in our hospital, which may not be representative enough. We look forward to a multi-center study in the future to reach more comprehensive conclusions.

## CONCLUSION

Motor imagery therapy (MIT) based on fNIRs technology has important clinical value in rehabilitation of upper limb function after cerebral infarction, boasting a variety of effects, such as improving the curative effect, ameliorating blood oxygen in brain tissue, and promoting the rehabilitation of upper limb function.

### Authors’ Contributions:

**YD & FH:** Designed this study, prepared this manuscript, are responsible and accountable for the accuracy and integrity of the work;

**YZ:** Collected and analyzed clinical data, significantly revised this manuscript.
